# Abundant bacteria shaped by deterministic processes have a high abundance of potential antibiotic resistance genes in a plateau river sediment

**DOI:** 10.3389/fmicb.2022.977037

**Published:** 2022-11-04

**Authors:** Yuhong Zhao, Hui Lin, Yi Liu, Ying Jiang, Weihong Zhang

**Affiliations:** ^1^Tibet Agricultural and Animal Husbandry University, Nyingchi, China; ^2^State Key Laboratory for Managing Biotic and Chemical Threats to the Quality and Safety of Agro-products, Institute of Environment Resource, Soil and Fertilizers, Zhejiang Academy of Agricultural Sciences, Hangzhou, China; ^3^Key Laboratory of Aquatic Botany and Watershed Ecology, Wuhan Botanical Garden, Chinese Academy of Sciences, Wuhan, China; ^4^University of Chinese Academy of Sciences, Beijing, China

**Keywords:** antibiotic resistance gene, potential hosts, plateau river sediment, community assembly, abundant bacteria

## Abstract

Recent research on abundant and rare bacteria has expanded our understanding of bacterial community assembly. However, the relationships of abundant and rare bacteria with antibiotic resistance genes (ARGs) remain largely unclear. Here, we investigated the biogeographical patterns and assembly processes of the abundant and rare bacteria from river sediment at high altitudes (Lhasa River, China) and their potential association with the ARGs. The results showed that the abundant bacteria were dominated by *Proteobacteria* (55.4%) and *Cyanobacteria* (13.9%), while the *Proteobacteria* (33.6%) and *Bacteroidetes* (18.8%) were the main components of rare bacteria. Rare bacteria with a large taxonomic pool can provide function insurance in bacterial communities. Spatial distribution of persistent abundant and rare bacteria also exhibited striking differences. Strong selection of environmental heterogeneity may lead to deterministic processes, which were the main assembly processes of abundant bacteria. In contrast, the assembly processes of rare bacteria affected by latitude were dominated by stochastic processes. Abundant bacteria had the highest abundance of metabolic pathways of potential drug resistance in all predicted functional genes and a high abundance of potential ARGs. There was a strong potential connection between these ARGs and mobile genetic elements, which could increase the ecological risk of abundant taxa and human disease. These results provide insights into sedimental bacterial communities and ARGs in river ecosystems.

## Introduction

Rivers play a vital and irreplaceable role in the process of human civilization and global biogeochemical cycling. Due to development of human society and rapid economic growth, river pollution has become a critical challenge (Shao et al., 2006; Wang et al., 2019). Antibiotic resistance genes (ARGs) and antibiotic-resistant bacteria (ARB) are recognized as emerging contaminants (Cosgrove, 2006; Amarasiri et al., 2020). Environmental pollution due to factors such as heavy metals may accelerate the enrichment and evolution of ARB and ARGs and increase the risk of transmission of the environmental resistome to humans (Yang et al., 2018). Bacterial community composition is a vital factor affecting the distribution of ARGs. For example, *Cyanobacteria* blooms promote the diversity of ARGs in aquatic ecosystems (Zhang et al., 2020). The change in the bacterial community promotes the improvement of ARGs in the chlorination process of drinking water (Jia et al., 2015). These studies on the correlation between bacterial community and ARGs were conducted at the overall level of the community. However, microbial communities in nature are comprised of a large number of species, while few of these species are abundant, and a large number of species are often called the “rare biosphere” (Sogin et al., 2006; Jiao et al., 2017). To date, we still know little about how spatial variation in ARG composition relates to bacterial taxonomic composition (i.e., abundant bacteria or rare bacteria) in a river continuum.

Abundant and rare bacteria in sediments are major participants in the biogeochemical cycle of rivers (Jia et al., 2015; Goldman et al., 2017). It is the core goal of community ecology to reveal the basic mechanism of the generation and maintenance of river microbial community diversity, and some interesting patterns have been discovered. For example, the physicochemical properties (such as pH, heavy metals content, and nutritional status) and spatial distribution (such as horizontal geographic distribution and vertical altitude distribution) were important drivers of the unique biogeographic patterns of microbial communities (Chen et al., 2020b; Wang Y. et al., 2020; Wang et al., 2021). However, there has been little consistency in the studies so far due to the heterogeneity of the river ecosystem.

In recent years, pollutants from industry and life have entered the water of the Lhasa River and caused a certain degree of pollution to the water quality. The changes in microbial community diversity and structure can indirectly or/and directly affect the aquatic ecological function, which is a comprehensive and sensitive index of environmental quality in the aquatic ecosystem (Long et al., 2021). The main objective of this study was to examine the biogeographical patterns and assembly processes of the abundant and rare bacteria in sediment and the potential association with the ARGs in the sediment of the Lhasa River. Therefore, 16S rRNA gene sequencing and qPCR reaction were used to analyze the sediments of the Lhasa River to determine the adaptation mechanism of microorganisms and resistance genes in the sediments. We hope this study could provide new insights into sedimental bacterial communities and ARGs in river ecosystems.

## Materials and methods

### Study sites and sample collection

Lhasa River (90.08–93.33°E, 29.33–31.25°N) known as the “Water Tower of Asia” is located in the Qinghai-Tibetan Plateau and is one of the highest rivers in the world (Qu et al., 2019). The Lhasa River basin is about 568 km long from east to west, and the altitude is between 3,570 and 5,200 m above sea level. More than 70% of the population of the Lhasa River Basin is concentrated from Mozhugongka county to Qushui county. Therefore, we set up 10 sampling sites along the Lhasa River from the Mozhugongka to the Qushui county with detailed geographic information on the sampling sites (Supplementary Table S1; Supplementary materials). Surface sediment (0–5 cm) was collected from each site in September 2019 using a stainless-steel core sampler. Three sub-samples were collected from each site, mixed as one sample, kept in a car refrigerator, transported to the laboratory, and stored at −80°C before 16S rRNA gene sequencing. The contents of Cr, Co, Cu, Zn, As, Cd, Hg, and Pb in sediment were detected by inductively coupled plasma mass spectrometer (ICP-MS, X Series 2, Germany). Detailed data and measurement methods are shown in Supplementary Table S2. Detailed information about sediment physicochemical properties (temperature, pH, salinity, and conductivity) and nutrients [total nitrogen (TN) and total carbon (TC)] are shown in Supplementary Table S3.

### 16S rRNA gene sequencing

Genomic DNA of the bacterial community from each site was extracted using a bacterial DNA Extraction Kit (Tiangen Biotech, Inc., Beijing, China) according to the manufacturer’s protocols (Zhang et al., 2021a). The DNA served as a template for PCR amplification of the V4 region of 16S rRNA using the primer set 515F/806R (Caporaso et al., 2011; Walters et al., 2016). The sequencing library was set up when the amplicons of 16S rRNA were purified, and Ion S5™XL of Thermofisher was used in the sequencing. The raw fastq data were quality-filtered by low-quality parts and chimeric sequences to get clean reads (Martin, 2011; Rognes et al., 2016). The clean reads were clustered into operational taxonomic units (OTUs) at the 97% similarity level using Uparse (Edgar, 2013). Since this study only focused on bacteria, we deleted all OTUs that did not belong to bacteria. The MUSCLE method and the SSU rRNA database of silva132 were used for the annotation species analysis (Wang et al., 2007; Quast et al., 2013). We followed a previously reported method (Wemheuer et al., 2020) and applied Tax4Fun to reveal the functional and redundancy index (FRI) of the sequenced bacterial genome.

### Analysis of ARGs in the sediment from the Lhasa River

A total of 23 ARGs and the 16S rRNA gene were selected to investigate the distribution of ARGs in the sediment from the Lhasa River. Herein, the representative ARGs in the environment and clinically important ARGs were taken into account based on the potential ecological risks and threats to human health (Zhang et al., 2021a; Jiang et al., 2021b). The 23 ARGs including seven major classes of antibiotic-related ARGs, which were the colistin (*mcr*-1, *mcr*-3, and *mcr*-7), beta-lactam (*bla*_CTX-M-32_, *bla*_NMD-1_, *bla*_CMY_, *bla*_CTX-M_, and *bla*_TEM_), aminoglycosides (*aad*A, *str*B, and *arm*A), macrolide (*ere*A, *ere*B, and *mph*A), quinolones (*qnr*A, *qnr*B, and *qnr*S), sulfonamides (*sul*1, *sul*2, and *sul*3), and tetracycline (*tet*A, *tet*M, and *tet*X) resistance genes, respectively. Besides, the transposase gene (*tnp*A) and class 1 integron-integrase gene (*int*I1) were selected to investigate the transfer or propagation of ARGs in the Lhasa River sediment. Detailed information on the primers and their corresponding target genes was given in Supplementary Table S4. The qPCR reaction in a 10 μl reaction volume was performed according to the denaturation at 95°C for 30 s, followed by the thermal cycles of qPCR consisting of 40 cycles of 10 s at 95°C, 30 s annealing at 55°C, and 1 min extension at 72°C. The relative abundances of ARGs and mobile genetic elements (MGEs) were calculated using the 2^−ΔCT^ method [Equations (1) and (2); Zhang et al., 2021a].


(1) $$△C_{T}=C_{T\left(\mathrm{ARG}\right)}-C_{T\left(16S\;\text{rRNA gene}\right)}$$

The relative abundance of


(2) $$\mathrm{ARG}={2^{-△C}}_{T}$$

### Statistical analysis

Previous studies have generally defined OTUs at the regional level with average relative abundances >0.10% as “abundant,” those with average relative abundances <0.01% as “rare” and those in between as “intermediate” (Jiao and Lu, 2020a; Wan et al., 2021a; Zhang et al., 2021b; Zhang Y. et al., 2021). However, this definition is not suitable for our data because the total abundance of these abundant bacterial OTUs in some samples was lower than 50% accounting for this sample’s total reads. Similarly, some previous studies also defined OTUs at the regional level with average relative abundances >0.05% as “abundant” (Jiao et al., 2017; Hou et al., 2020). Thus, across all sediment, the average relative abundance of OTUs above 0.05% was defined as abundant bacteria, while the average relative abundance of OTUs below 0.01% was regarded as rare bacteria. The remainder OTUs (0.01–0.05%) were deemed as “intermediate.” The community similarity (1-Bray–Curtis distance) and phylogenetic similarity (1-βNMTD) of abundant and rare bacteria were calculated based on taxonomic distance and phylogenetic distance, respectively. Then, the distance-decay relationship (DDR) was used to reveal the responses of community similarity and phylogenetic similarity to horizontal (geographic distance) and vertical (altitude distance) spatial distribution and environmental heterogeneity (Bray-Curtis distance). The network was constructed by Spearman correlation and visualized *via* Gephi software (0.9.1; Gephi, WebAtlas, France). We identify the contribution of different assembly processes of abundant and rare bacteria in the Lhasa River sediments *via* applying a null model analysis by Stegen et al. (2013).

## Results

### Composition and distribution of abundant and rare bacteria

**Figure 1:**
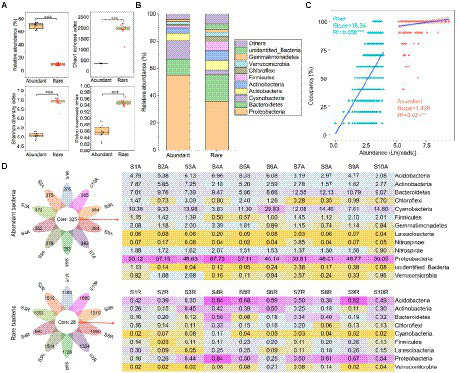
Alpha diversity and composition of abundant and rare bacteria in sediment from the Lhasa River. **(A)** Alpha diversity of abundant and rare bacteria in sediment. **(B)** The composition of abundant and rare bacteria in sediment. **(C)** Abundance–occupancy relationship of abundant and rare bacteria in sediment. **(D)** The number and composition of shared operational taxonomic units (OTUs) in sediment. Asterisks denote significance (^**^p < 0.01; ^***^p < 0.001).

The relative abundance of abundant bacteria (mean = 69.6%) was higher than rare ones (10.5%; Figure 1A). Conversely, the Chao1 richness (381.6), Shannon diversity (5.08), and Pielou evenness (0.86) of abundant bacteria were lower than the rare ones (1937.5, 6.88, and 0.95, respectively; Figure 1A). At the bacterial phylum level, abundant bacteria were dominated by *Proteobacteria* (55.1%), *Cyanobacteria* (13.8%), and *Bacteroidetes* (11.6%), while the *Proteobacteria* (36.1%), *Bacteroidetes* (19.3%), and *Actinobacteria* (7.17%) were the main components of rare bacteria (Figure 1B). Abundance-occupancy relationships showed that rare bacteria possessed stronger positive correlations than abundant bacteria (Figure 1C). Meanwhile, abundant bacterial taxa had a wider distribution than the rare bacterial taxa. The petal diagram showed that abundant bacteria had 325 OTUs that persisted in all sediments, while rare bacteria only had 28 OTUs (Figure 1D). Even these persistent abundant and rare bacteria had obvious differences in spatial distribution.

**Figure 2:**
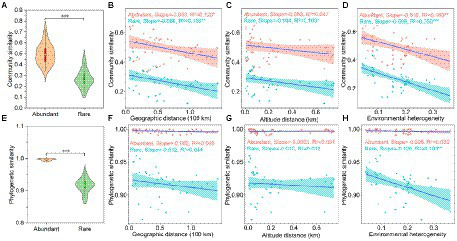
Beta-diversity patterns of taxonomic and phylogenetic for both abundant and rare bacteria in sediment from the Lhasa River. **(A)** Community similarity (1—Bray-Curtis distance) of abundant and rare bacteria. **(B–D)** Relationship of community similarity for both abundant and rare bacteria with the geographical distance, altitude distance, and environmental heterogeneity, respectively. **(E)** Phylogenetic similarity (1—βMNTD) of abundant and rare bacteria. **(F–H)** Relationship of phylogenetic similarity for both abundant and rare bacteria with the geographical distance, altitude distance, and environmental heterogeneity, respectively. Asterisks denote significance (^*^p < 0.05; ^**^p < 0.01; and ^***^p < 0.001).

The community similarity (Figure 2A) and phylogenetic similarity (Figure 2E) of abundant bacteria were higher than rare bacteria, indicating that the rare bacteria had more taxonomic and phylogenetic variation than the abundant bacteria. Furthermore, the community similarity for abundant and rare bacteria had significantly positive correlations with their corresponding phylogenetic similarity, and the correlations of rare bacteria were stronger than that of abundant bacteria (Supplementary Figure S1), indicating that the phylogeny of these abundant and rare bacteria had different sensitivities to environmental changes.

The DDR showed that the community similarity of both abundant and rare bacteria significantly decreased with the increased geographical distance (Figure 2B). Interestingly, the effect of geographical distance on the community composition of rare bacteria (*R*^2^ = 0.15) was greater than that of abundant bacteria (*R*^2^ = 0.12), whereas the community composition of abundant bacteria (Slope = −0.083) had more community turnover with increased of geographical distance. Besides, the composition of rare bacteria was also significantly affected by altitude in biogeographic patterns, and the community similarity significantly decreased with the increased altitude distance (Figure 2C). Similarly, the effect of environmental heterogeneity on rare bacteria (*R*^2^ = 0.35) was greater than that of abundant bacteria (*R*^2^ = 0.18). Rare bacteria (Slope = −0.609) had more community turnover with the increase in environmental change (Figure 2D). Specifically, the taxonomic composition of rare bacteria was significantly affected by heavy metals, such as Cu, Zn, Cd, and As, whereas only the composition of abundant bacteria was significantly affected by Cu (Supplementary Table S5). Especially, Cu had more influence on the taxonomic composition of rare bacteria than abundant bacteria (Supplementary Table S5). However, the phylogenetic similarity for abundant and rare bacteria did not decrease significantly with the increased geographical distance and altitude distance (Figures 2F,G). Only the phylogenetic similarity of rare bacteria was significantly affected by environmental heterogeneity (Figure 2H). The different responses of abundant and rare bacteria to geographic and environmental factors in the taxonomic and phylogenetic composition may indicate the presence of had distinct community ecological assembly processes.

### Community assembly processes of abundant and rare bacteria in the sediment

**Figure 3:**
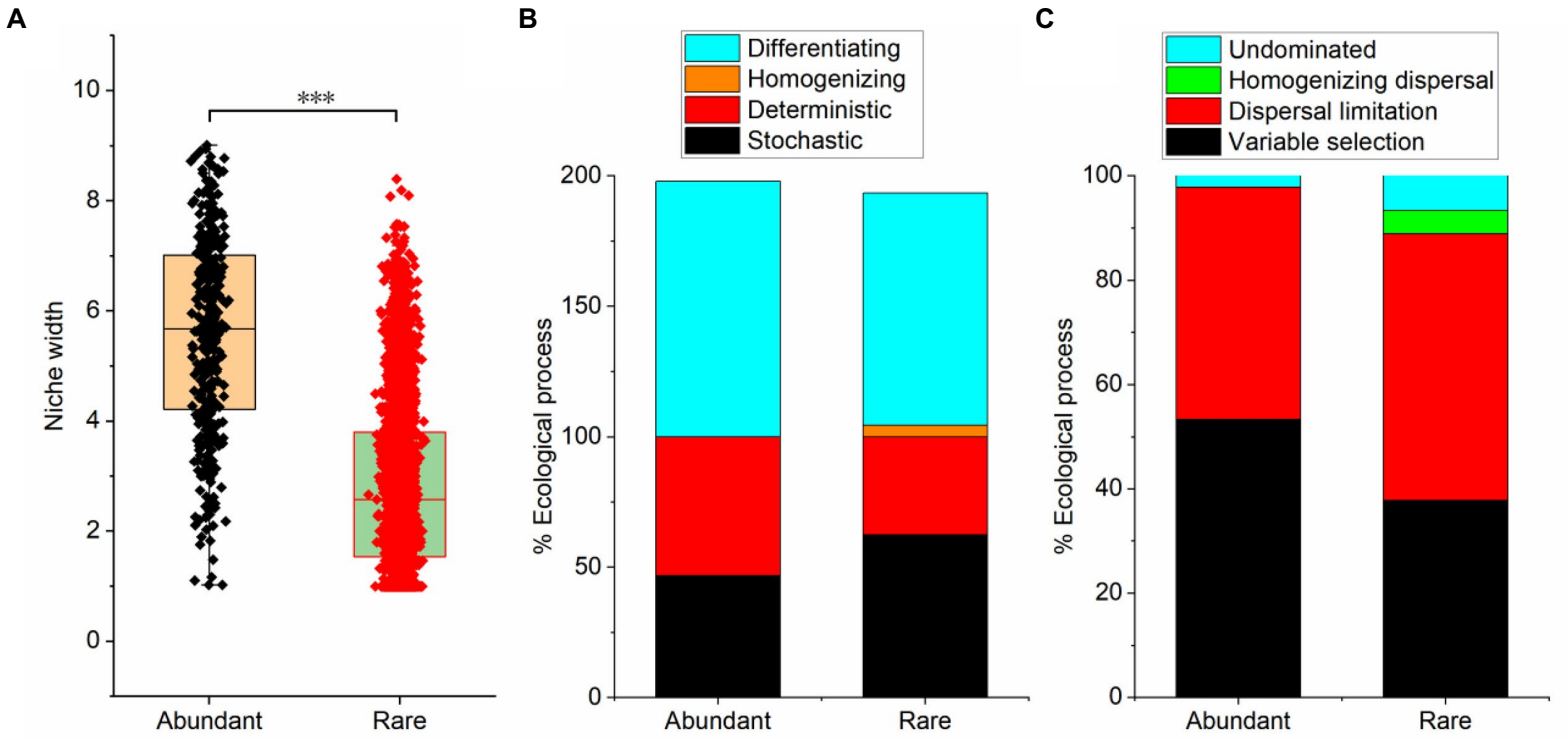
Niche width **(A)** and community assembly processes **(B,C)** of abundant and rare bacteria in sediment from the Lhasa River. Stochastic = Dispersal limitation + Homogenizing dispersal + Undominated processes; Deterministic = Variable selection + Homogeneous selection; Homogenizing = Homogeneous selection + Homogenizing dispersal; and Differentiating = Variable selection + Dispersal limitation.

Although the niche width of abundant bacteria (mean = 5.58) was higher than rare bacteria (2.80), the niche of the abundant bacteria showed higher differentiation (Figure 3A). Results from the null model showed that the differentiating was the dominant process for both abundant (99.8%) and rare bacteria (88.9%) assembly, while the homogenizing process (4.44%) had little impact on rare bacteria assembly (Figure 3B). Additionally, the stochastic process (62.2%) was the main assembly pattern of rare bacteria in the sediment of the Lhasa River, followed by the deterministic process (37.8%). The deterministic process (53.3%) dominated the assembly of abundant bacteria, followed by the stochastic process (46.7%). The results show that the contribution of the stochastic and deterministic process for the assembly of abundant and rare bacteria in the sediment of the Lhasa River was different. Mantel tests suggested that the βNTI values of both abundant and rare bacteria had a significant correlation with the geospatial factor (latitude; Supplementary Table S6), indicating that the community assembly of abundant and rare bacteria may be affected by latitude. Furthermore, the βNTI values of abundant bacteria significantly correlated with pH, conductivity, and heavy metal (Cd; Supplementary Table S6). The results of the null model further suggested that the variable selection (53.3%) was the dominant assembly process of abundant bacteria, whereas the dispersal limitation (51.1%) was the dominant assembly process of rare bacteria (Figure 3C). These results suggested that the assembly of abundant bacteria was susceptible to the environmental selection, while the assembly of rare bacteria was susceptible to geospatial factors.

### Co-occurrence patterns of abundant and rare bacteria

**Figure 4:**
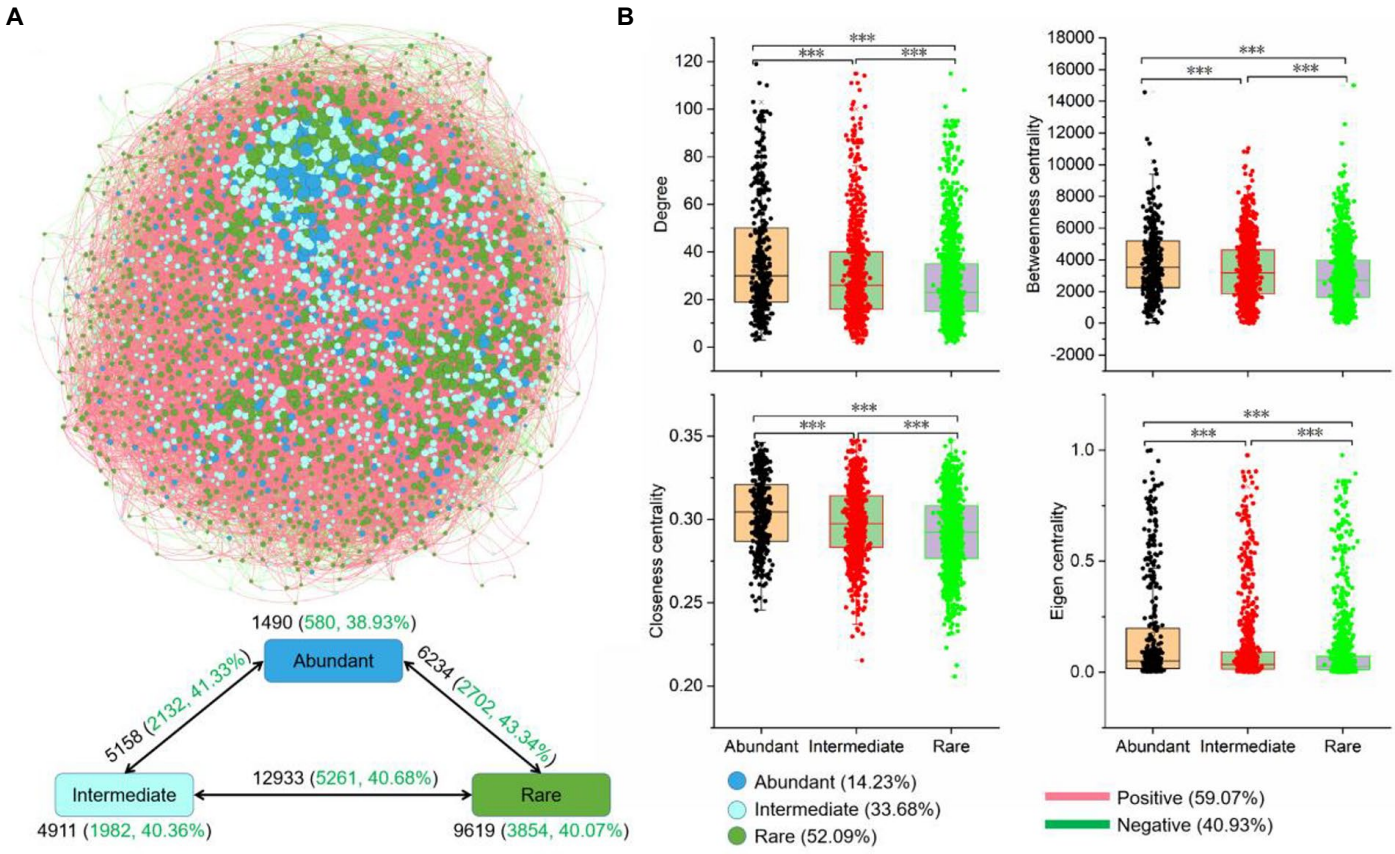
Co-occurrence network of abundant and rare bacteria in the sediment of the Lhasa River. **(A)** The network analysis showed the intra-associations within each bacterial taxa and inter-association between different bacterial taxa. OTUs occurred in more than half of samples were used for network analysis. A connection based on a strong (|*r*| > 0.8) and significant (*p* < 0.01) correlation *via* Spearman. The size of each node is proportional to the degree. Numbers outside and inside parentheses represent total edge numbers and negative edge numbers and their ratio, respectively. **(B)** Comparison of node-level topological features among three different bacterial taxa. Asterisks denote significance (^***^p < 0.001).

A metacommunity network was conducted based on the strong (|*r*| > 0.8) and significant (*p* < 0.01) Spearman correlations to explore the co-occurrence patterns of the sedimental microbial communities of the Lhasa River (Figure 4A). The network consisted of 2,699 nodes linked by 40,344 edges. Degree, Betweenness centrality, Closeness centrality, and Eigen centrality of the network within abundant bacteria were significantly higher than within rare bacteria (Figure 4B), indicating that the abundant bacteria played an important role in maintaining community structure. Abundant bacteria interacted more with other bacterial taxa than within themselves (Figure 4A). Although the number of positive correlation edges (59.1%) was higher than that of negative correlation edges (40.9%) in the co-occurrence network of whole bacteria in sediment, the proportion of negative correlation was different within and between the different bacterial taxa. For example, the proportion of negative correlation within bacterial taxa was lower than between these bacterial taxa, suggesting there may be stronger competition between different bacterial taxa than that within these bacterial taxa. Further, the proportion of negative correlation within rare bacteria (40.1%) was higher than within abundant bacteria (38.9%), indicating that there may be stronger competition within rare bacteria than within abundant bacteria.

### Potential function analysis of abundant and rare bacteria

**Figure 5:**
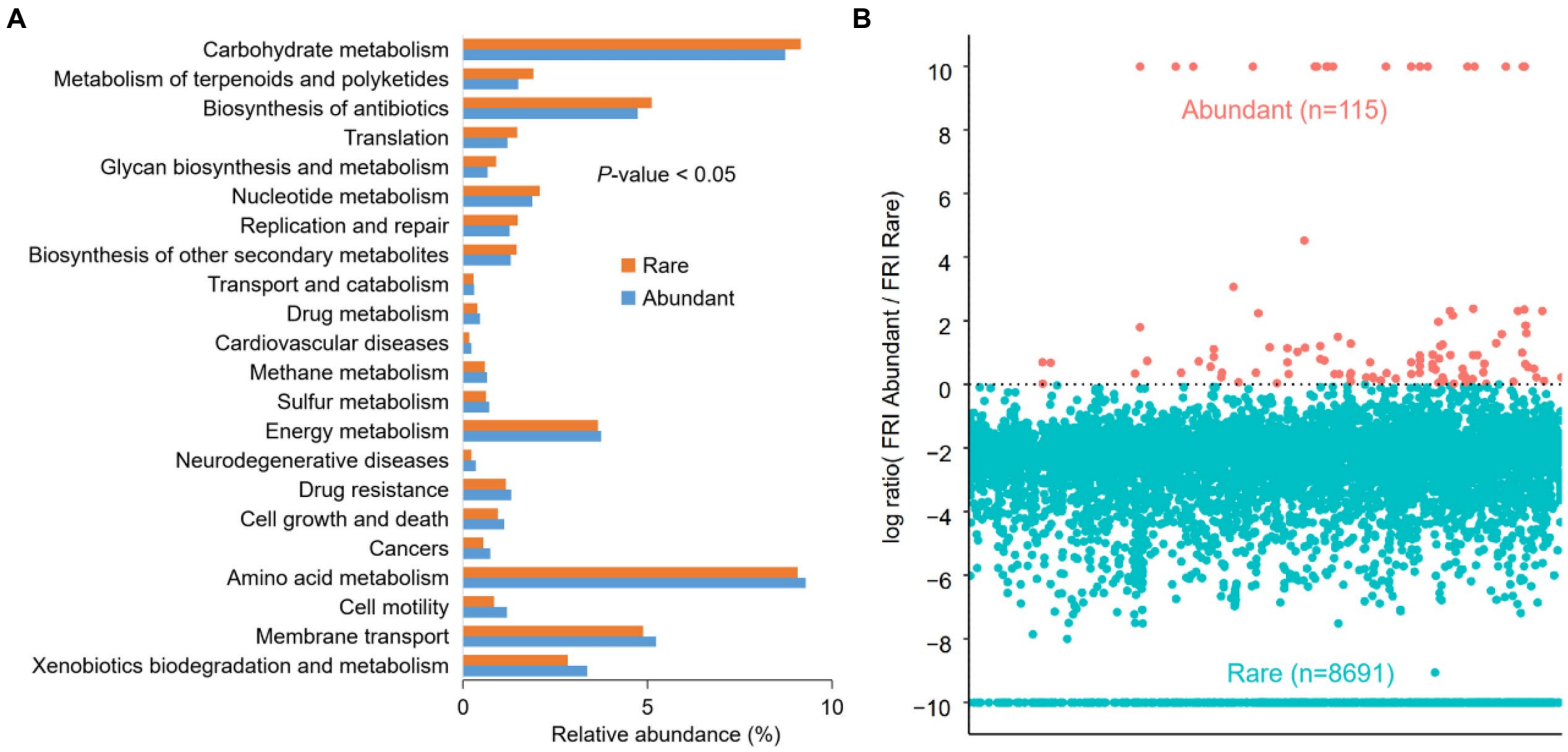
Comparison of functional differences **(A)** and functional redundancy **(B)** between rich and rare groups in sediments of the Lhasa River.

When compared to rare bacteria, abundant bacteria not only had the highest abundance of metabolic pathways of potential drug resistance (such as antimicrobial and antineoplastic resistance), chemical structure transformation maps, cell growth and death, cell motility, and xenobiotics biodegradation and metabolism but also had the more potential pathogenic potential of human disease (such as infectious diseases, etc.) in all predicted functional genes (Figure 5A). However, abundant taxa had weak global and overview maps (such as biosynthesis of antibiotics and carbon metabolism), carbohydrate metabolism, metabolism of terpenoids and polyketides, nucleotide metabolism, glycan biosynthesis and metabolism, and biosynthesis of other secondary metabolites. The function redundancy index (FRI) of abundant bacteria (122) was lower than that of rare bacteria (8,878; Figure 5B), indicating that the probability of potential function loss of rare bacteria after the disturbance was lower than that of abundant bacteria.

### Composition and distribution of ARGs

**Figure 6:**
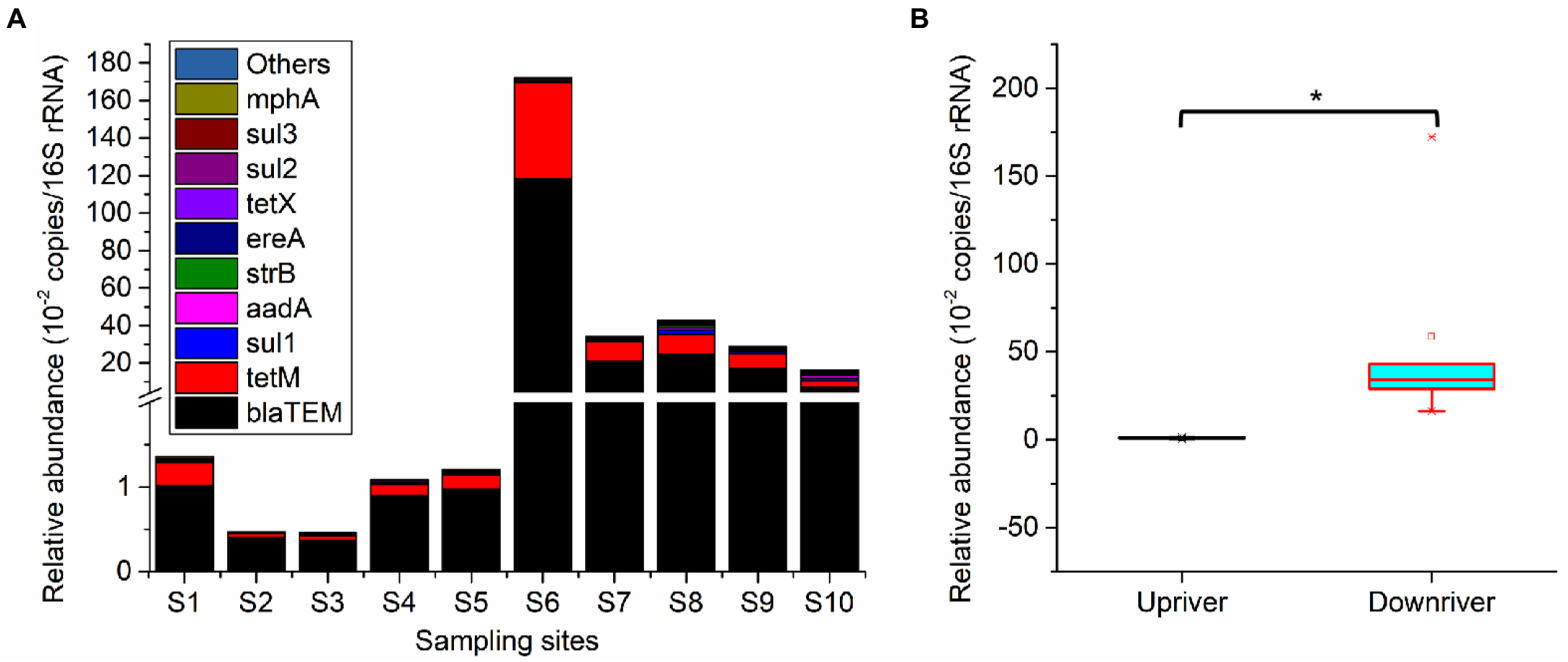
Main composition of the sedimental ARGs of the Lhasa River. Upriver including the sediment samples from sampling site S1 to S5, and downriver including the sediment samples from sampling site S6 to S10. “*” showed a significant difference at the 0.05 level.

A total of 20 ARGs were detected in sediment samples of the Lhasa River, which including colistin (*mcr*-1, *mcr*-3, and *mcr*-7), beta-lactam (*bla*_CTX-M-32_, *bla*_CMY_, *bla*_CTX-M_, and *bla*_TEM_), aminoglycosides (*aad*A and *str*B), macrolide (*ere*A, *ere*B, and *mph*A), quinolones (*qnr*A, *qnr*B, and *qnr*S), sulfonamides (*sul*1, *sul*2, and *sul*3), and tetracycline (*tet*M and *tet*X) resistance genes, respectively (Figure 6A). However, *bla*_NMD-1_, *arm*A, and *tet*A were not detected in any sediment sample. The total relative abundance of ARGs ranged from 4.60 × 10^−3^ to 1.72 copies per 16S rRNA, indicating that the ARGs were widely distributed in the sediment of the Lhasa River. The *bla*_TEM_ was the only ARG detected in all sediments and was the most abundant ARGs (mean relative abundance was 1.92 × 10^−1^ copies per 16S rRNA), followed by the *tet*M, *sul*1, and *aad*A. Besides, *aad*A, *str*B, *sul*1, *sul*2, and *tet*M were also detected in all sediments. Among the 10 sediment samples, the total relative abundance of ARGs at S6 was significantly higher than those of other locations. The total relative abundance of ARGs downstream from Lhasa (from S6 to S10) was significantly higher than those upriver from Lhasa (from S1 to S5), suggesting that human activities may promote the accumulation of ARGs in the sediments of the Lhasa River (Figure 6B).

### Potential hosts and co-occurrence patterns of ARGs in sediment

**Figure 7:**
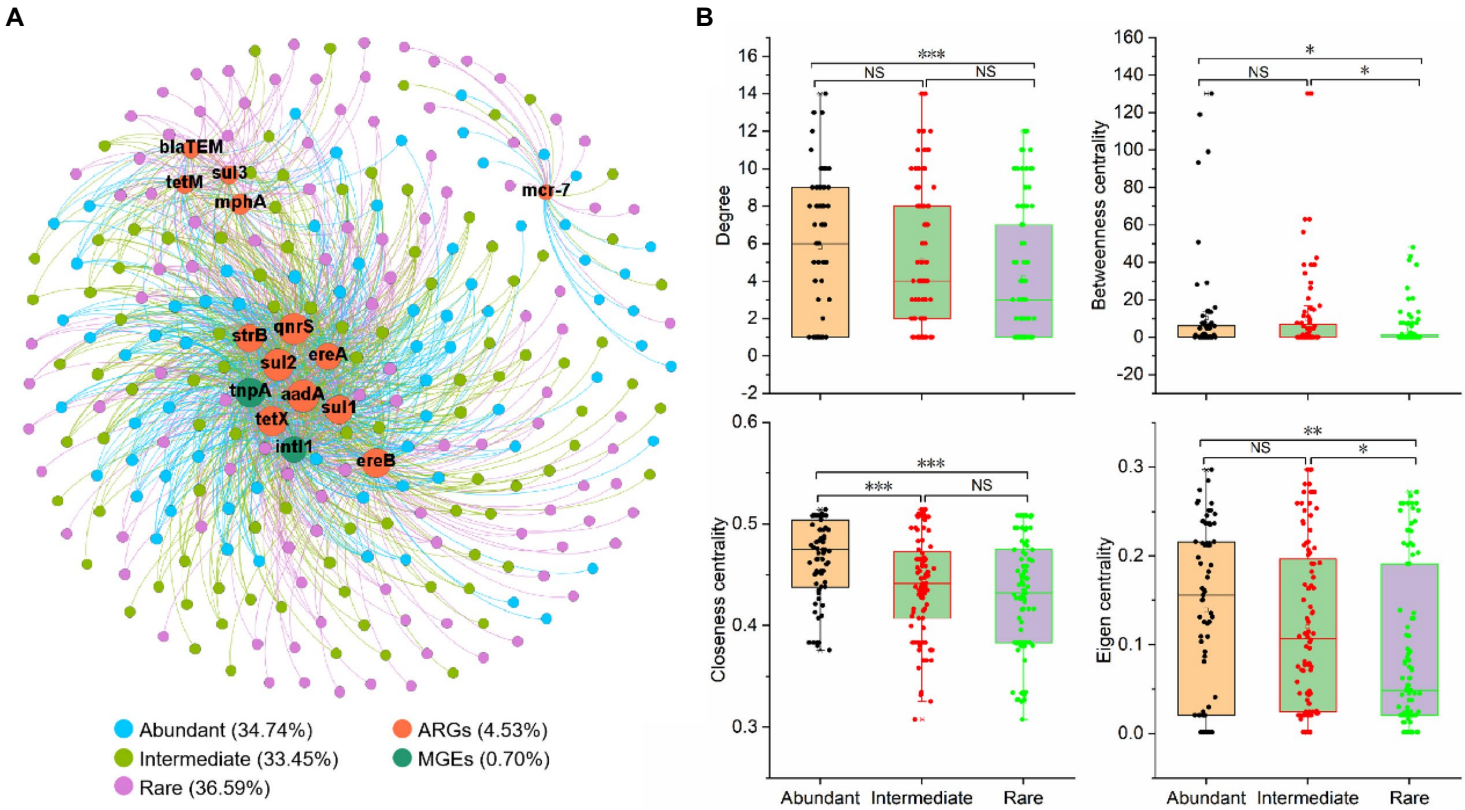
Co-occurrence patterns of ARGs and their potential hosts in the sediment of the Lhasa River. **(A)** Network analysis showed the co-occurrence patterns of ARGs and their potential hosts. The percentages were of these taxa OTUs or genes that accounted for total OTUs or genes in networks. OTUs and ARGs occurred in more than half of the samples were used for network analysis. A connection based on a strong (*r* > 0.8) and significant (*p* < 0.01) correlation *via* Spearman. **(B)** Major network topological properties of co-occurrence patterns of ARGs and their potential hosts. Asterisks denote significance (NS, *p* ≥ 0.05; ^*^p < 0.05; ^**^p < 0.01; and ^***^p < 0.001).

Network analysis showed that more members of rare bacteria (36.5%) were the potential host of ARGs (Figure 7A). An ARG may have more potential hosts, such as the potential hosts of *mcr*-7 belonging to both abundant and rare bacteria. However, network topology features showed that abundant bacteria rather than rare bacteria had stronger connectivity and centrality, indicating that abundant bacteria may be the potential host of more ARGs (Supplementary Figure S2). Besides, the relative abundance of abundant bacteria (15.5%) in the whole bacterial community was higher than that of rare bacteria (0.57%; Supplementary Figure S3). This also suggested that the abundant bacteria were the main potential host of ARGs. The relative abundance of ARGs and their potential hosts downstream was higher than upstream, suggesting that urbanization may promote the occurrence of ARGs and their potential hosts.

Furthermore, the network results showed coexistence patterns among some ARGs, such as *bla*_TEM_ had a significant correlation with the *str*B, *mph*A, *sul*3, and *tet*M. More important, *transposase gene tnpA* had a significant correlation with the *aad*A, *str*B, *ere*A, *ere*B, *qnr*S, *sul*1, *sul*2, and *tet*X. *int*I1 had a significant correlation with the *aad*A, *str*B, *ere*B, *qnr*S, *sul*1, *sul*2, and *tet*X. These results suggested that some ARGs (*aad*A, *str*B, *ere*B, *qnr*S, *sul*1, *sul*2, and *tet*X) in the sediment of the Lhasa River may co-exist on MGEs, which may increase the risk of transmission of these ARGs in aquatic ecosystems.

## Discussion

Bacterial communities are the foundation of every ecosystem on earth. They are often composed of abundant bacterial taxa with fewer species and rare bacteria with more species (Pedrós-Alió, 2012; Hou et al., 2020; Zhang et al., 2021b). Research on abundant and rare bacteria has expanded our understanding of bacterial community structure, but the relationships of abundant and rare bacteria with ARGs remain largely unclear. Revealing the dominant host bacteria (e.g., abundant or rare bacteria) of ARGs and assembly processes provide a good hold on the potential risks of ARGs. Therefore, we investigated the composition of abundant and rare bacteria and their relationship with ARGs. We also characterized the ecological assembly mechanism of sedimental abundant and rare bacteria in the Lhasa River, China.

### Rare taxa can serve as function insurance of sediment bacterial communities

In this study, rare bacteria with low relative abundance were found to have high richness and diversity (Figure 1A), which was consistent with findings of the studies on the sediment of Erhai Lake (Zhang et al., 2021b) and Hangzhou Bay (Dai et al., 2016). Although rare bacteria did not dominate the taxonomic community, they may still play an important role in maintaining the bacterial community’s stability in the Lhasa River sediment due to their large taxonomic pool. Because the more members of the rare bacteria, the stronger the buffering effect of their functional composition on environmental variation (Schindler et al., 2010). Previous studies showed that functional redundancy could protect microbial communities by maintaining ecosystem function homeostasis (Liang et al., 2020). Our study found that rare bacteria had stronger functional redundancy than abundant ones (Figure 5B). Besides, rare bacteria with high functional redundancy show a stronger adaptation to anthropogenic disturbances (Wan et al., 2021b). Thus, rare bacteria can serve as an insurance source for the function of sediment bacterial communities in the Lhasa River during external disturbance.

### Biogeographical patterns of abundant and rare bacteria in the sediment

Our study found obvious differences in diversity, taxonomy composition, and phylogenetic composition between abundant and rare bacteria. Even the persistent abundant and rare bacteria showed different biogeographical patterns. The number of spatial persistence existing OTUs in abundant bacteria was outdistanced than that in rare ones, which was consistent with the spatial persistence existing pattern of rare and abundant bacteria in the sediment from Erhai Lake (Zhang et al., 2021b). Furthermore, this study found that the community similarity of abundant bacteria stands out from rare bacteria (Figure 2A), suggesting the species composition of rare bacteria was more susceptible to geographical and environmental filters. Some studies found that the stronger spatial variation within the microbial community, the more susceptible it is to environmental change (Wan et al., 2021b; Zhang et al., 2021b). It also may be foreshadowed that rare bacteria in sediments from the Lhasa River were more susceptible to environmental changes.

Geographic distance and environmental heterogeneity are abiotic factors that govern bacterial community assemblage (Gao et al., 2019; Langenheder and Lindström, 2019). This study found that abundant and rare bacteria had complicated responses to geographical and environmental differences. The spatial turnover of the bacterial community has been reported to be related to dispersal limitations (Wang et al., 2013; Lewthwaite et al., 2017). Our study found that rare and abundant bacteria had a more significant variation in horizontal spatial distribution because their community similarity decreased with the increase in geographical distance, which was similar to the biogeographical pattern of river microorganisms reported previously (Chen et al., 2020a,b; Wang Y. et al., 2020). This study also found that altitude was another important spatial factor that affects the community turnover of rare bacteria in the sediment. The *R*^2^ value of DDR showed that the geographical distance, altitude distance, and environmental heterogeneity had greater effects on rare bacteria than on abundant bacteria, showing that rare bacteria rather than abundant bacteria were more susceptible to environmental changes. The slope of DDR showed that the effects of altitude and environmental heterogeneity on the spatial turnover of rare bacteria surpass abundant bacteria. These results portend that geographic and environmental factors together shaped the unique biogeographic pattern of sediment abundant and rare bacteria in the Lhasa River. This also means the greater impact of geographic and environmental factors on rare bacteria resulting in the community similarity of rare bacteria far below abundant bacteria.

### Potential associations of bacterial communities

Association among the microbe-microbe is an essential biotic factor in the assembly processes of microbial communities except for the abiotic factor (geographic and environmental selection; Nemergut et al., 2013; Zhang et al., 2021b). In the community ecological assembly processes, network analysis could provide new insights into the associations within individual bacterial taxon and linkages between different bacterial taxa (Xue et al., 2018; Zhang et al., 2021b). The nodes in networks with high connectivity may play a crucial role in protecting the structural stability of the bacterial community (Xue et al., 2018). This study found that the connection within the abundant bacteria significantly overtopped rare bacteria, indicating that abundant bacteria may play an irreplaceable role in maintaining bacterial community structure. Furthermore, the positive interaction links in the network are mainly considered cooperative relationships among microbial members, while the negative interaction links are mainly thought of as competitive relationships among microbial members (Faust et al., 2012). Cooperation among bacterial members helps improve the resilience of bacterial communities to respond to changing environments (Xue et al., 2018). Cooperation among the abundant bacteria was more than among rare bacteria, which may be an important reason for the widespread of abundant bacteria in the sediment of the Lhasa River.

### Deterministic process was the dominant assembly process in abundant bacterial taxa

Traditional niche theory generally agrees that deterministic process mediated community structure is governed by species interaction (e.g., competition and mutualisms, etc.) and environmental variables (e.g., pH and temperature, etc.; Fargione et al., 2003; Zhou and Ning, 2017), whereas neutral theory assumes that community structure is shaped by limited dispersal and random fluctuations in species abundance (e.g., birth, death, and extinction, etc.; Chave, 2004; Zhou and Ning, 2017). Although deterministic and stochastic were generally accepted that occur simultaneously in the community assembly processes, their relative contribution to regulating community structure and biogeography is debatable (Zhou and Ning, 2017). This study showed that both deterministic and stochastic processes occur during the assembly of abundant and rare bacteria, which was consistent with previous studies (Zhou and Ning, 2017; Jiao and Lu, 2020b; Wan et al., 2021b; Zhang et al., 2021b). Among them, the deterministic process was the dominant assembly mechanism of abundant bacterial taxa, while the stochastic process was the dominant assembly mechanism of rare bacteria. One possible reason was that the high diversity of rare bacteria species allows them to occupy various ecological niches, while more rare species occurs spatial turnover in biogeographic distribution, which leads to the strong influence of stochastic processes on the assembly of rare bacterial taxa (Hou et al., 2020). Similarly, more persistent species from the abundant bacterial taxa were detected in the Lhasa River sediment, which may be one reason why the assembly processes of the abundant bacteria were more inclined to deterministic processes. Furthermore, environmental and spatial variables also seemed to control the biogeographic patterns and assembly of abundant and rare bacteria. Null model results showed that the variable selection was the dominant assembly process of abundant bacteria, followed by dispersal limitation. Conversely, the dispersal limitation was the main assembly process of rare bacteria, followed by variable selection. Moreover, the Mantel tests of geospatial and environmental factors against βNTI values also suggested that the βNTI values of abundant bacteria had a significant correlation with more geospatial and environmental factors (such as latitude, pH, conductivity, TN and TC ratio, and Cd), whereas βNTI of rare bacterial taxa only had a significant correlation with latitude. This may be a decisive reason why the abundant bacteria were more influenced by variable selection than rare bacteria.

### Urbanization increased the occurrence of ARGs in the Lhasa River sediment

Antibiotic resistance genes were widely distributed in the sediment of the Lhasa River, among which *bla*_TEM_, *aad*A, *str*B, *sul*1, *sul*2, and *tet*M were detected with 100%. Notably, *bla*_TEM_ was detected in all sediment samples with the highest abundance, consistent with *bla*_TEM_ in the surface sediments of Danjiangkou Reservoir (Jiang et al., 2021b). The *bla*_TEM_ was the clinically relevant ARG, which can be used as an indicator gene for ARG contamination caused by human activities (Narciso-da-Rocha et al., 2014; Stange et al., 2019). This also indicates that sediments of the Lhasa River were contaminated by ARGs related to human activities. Human activities have increased correspondingly with the decrease of the altitude of the Lhasa River, which also leads to the increase in the abundance of ARGs in the sediments. In contrast to a global survey that found that urbanization was strongly associated with lower rates of antibiotic resistance (Collignon et al., 2018), studies have reported that urbanization could promote the development of bacterial resistance to antibiotics in rivers (Peng et al., 2020; Liu et al., 2021). This study found that urbanization promoted the enrichment of ARGs, which was consistent with the results found in the Yarlung Tsangpo River (Liu et al., 2021). Therefore, more attention should be paid to the pollution of ARGs caused by urbanization on the watershed scale.

### Abundant bacterial taxa were the main potential hosts of ARGs

Bacterial antibiotic resistance is one of the most serious global threats to environmental safety and human health (Ashbolt et al., 2013; Roca et al., 2015; Zainab et al., 2020). *Cyanobacteria* were found to be a reservoir and source of ARGs (Wang Z. et al., 2020), which contributes to the diversity increase of ARGs in the aquatic ecosystem (Zhang et al., 2020). In our study, the relative abundance of *Cyanobacteria* was second only to that of *Proteobacteria* among the abundant bacterial taxa, but not in rare bacterial taxa. Previous studies also found that *Proteobacteria*, *Bacteroidetes*, and *Actinobacteria* were often antibiotic producers or have the ability to transform/metabolize (Manaia, 2017). *Proteobacteria*, *Bacteroidetes*, and *Actinobacteria* were the dominant phyla in both abundant and rare bacteria. Furthermore, abundant bacteria account for the highest proportion in whole bacterial communities, indicating abundant bacteria may be the main hosts of ARGs in the sediments from the Lhasa River. Function prediction results show that abundant bacteria not only had a strong pathogenic potential for human diseases but also had a strong potential for drug resistance. Environment ARGs could threaten human health by increasing pathogenic ARB, leading to inefficient or ineffective use of therapeutic antibiotics for humans (Pan et al., 2020; Jiang et al., 2021a). To date, we still know little about how spatial variation in ARGs composition relates to bacterial taxonomic composition (i.e., abundant bacteria or rare bacteria) in a river continuum. Therefore, we further explored the relationship of the ARGs with abundant bacteria and rare bacteria based on understanding the biogeographic patterns of ARGs.

The co-occurrence network is also a widely used as an important tool to explore the interaction between ARGs and their potential hosts (Chen et al., 2016; Peng et al., 2020). Important nodes in a network can be identified by network central location and high connectivity (Peng et al., 2020). In this study, the network analysis showed that the connectedness between the abundant bacteria and ARGs was higher than the rare ones (Figure 7B), indicating that the abundant bacteria may be the potential hosts of more ARGs. The relative abundance of these potential hosts belonging to abundant bacteria was also higher than that of rare bacteria. This may also be an important reason for the strong potential drug resistance in the abundant bacterial taxa. More importantly, ARGs in the environment lead to the rapid increase in the spread and number of ARB through horizontal gene transfer, which will also make antibiotic resistance an important and unavoidable global health problem affecting human health (Watts et al., 2017; Manyi-Loh et al., 2018; Kraemer et al., 2019; Yadav and Kapley, 2021). This study found that abundant bacteria not only had strong potential drug resistance but also have a high abundance of potential ARGs. There was a strong and significant correlation between these ARGs and MGEs, which could increase the ecological risk of abundant taxa and the potential for human disease.

Some potential limitations merit further discussion. Our analyses were focused on the abundant and rare bacterial taxa level, and we did not know the exact host bacteria for each ARG at the species level. Network results on the co-occurrence patterns between ARGs and bacterial taxa indicated the possible host information of ARGs. Therefore, further studies are needed to verify the ARG bacterial hosts at the species level. Abundant bacteria had a high abundance of metabolic pathways of potential drug resistance in all predicted functional genes may be due to their higher relative abundance in the whole community, but it also does not mean that rich taxa contain more ARGs than rare taxa. Therefore, further studies are needed to verify the ARG bacterial hosts at the species level.

## Conclusion

In this study, we investigated biogeographical patterns and assembly mechanisms of rare and abundant bacteria and revealed the potential association among the ARGs with abundant and rare bacteria from the Lhasa River sediment. The different and complex responses of abundant and rare bacteria to geospatial and environmental changes may be influenced mainly by deterministic and stochastic processes, respectively. Rare taxa can serve as function insurance of bacterial communities in the Lhasa River sediment. This shall provide novel insights to explain the assembly and biogeographical patterns of abundant and rare bacteria in the sediment. To our knowledge, this study was the first time to reveal that the abundant bacteria have a high abundance of potential ARGs in Plateau Rivers, with strong pathogenic potential for human diseases. In particular, abundant bacteria with potential ARGs were also maybe the main potential hosts for the presence of MGEs, which may increase the ecological risks of abundant bacteria. These results provide new insights into understand the ARGs’ association with abundant and rare bacteria in plateau river sediment. Given the importance of ARGs to the health of aquatic ecosystems, the findings of this study should be validated experimentally at the bacterial species level in more diverse freshwater and marine ecosystems.

## Data availability statement

The data presented in the study are deposited in the Sequence Read Archive (SRA) repository (https://submit.ncbi.nlm.nih.gov/subs/sra/), accession number PRJNA681935.

## Author contributions

YZ and WZ: conceptualization. YZ and YJ: methodology. YZ and HL: writing–original draft and validation. YL and HL: visualization. YL and YJ: software. WZ: writing–reviewing and editing. All authors contributed to the article and approved the submitted version.

## Funding

This work was supported by the National Natural Science Foundation of China (Grant numbers 31860151 and 32171648).

## Conflict of interest

The authors declare that the research was conducted in the absence of any commercial or financial relationships that could be construed as a potential conflict of interest.

## Publisher’s note

All claims expressed in this article are solely those of the authors and do not necessarily represent those of their affiliated organizations, or those of the publisher, the editors and the reviewers. Any product that may be evaluated in this article, or claim that may be made by its manufacturer, is not guaranteed or endorsed by the publisher.
