# Supplementary material for: Abundant bacteria shaped by deterministic processes have a high abundance of potential antibiotic resistance genes in a plateau river sediment
Source: Front Microbiol. 2022 Nov 4;13:977037. doi: 10.3389/fmicb.2022.977037 (PMC9672519; doi:10.3389/fmicb.2022.977037)
Supplement: Supplementary file 1 [file Data_Sheet_1.pdf]

Abundant bacteria shaped by deterministic was the main potential host of antibiotic resistance genes in river sediment

Weihong Zhang[a,b,1], Yuhong Zhao[c,1], Hui Lin [d], Yi Liu[a,b], Ying Jiang[a,b], Yuyi Yang[a,b*]

[a] Key Laboratory of Aquatic Botany and Watershed Ecology, Wuhan Botanical Garden, Chinese Academy of Sciences, Wuhan, 430074, China

[b] University of Chinese Academy of Sciences, Beijing, 100049, China

[c] Tibet Agricultural and Animal Husbandry College

[d] The Institute of Environment, Resource, Soil and Fertilizers, Zhejiang Academy of Agricultural Sciences, Hangzhou 310021, China

[1] The authors make equal contributions to this manuscript.

Corresponding author

Prof. Yuyi Yang

Wuhan Botanical Garden, Chinese Academy of Sciences, Lumo Road No.1, Wuchang District, Wuhan, China. Phone: +86 27 87700853, Fax: +86 27 87510251,

Email: yangyy@wbgcas.cn

Table S1. Information of sampling sites and sampling from the Lhasa River.

Sites	Longitude (E)	Latitude (N)	Altitude (m)	Samples
S1	92.19500000	29.70972222	4335	Sediment 1 (S1)
S2	91.76638889	29.87305556	3868	Sediment 2 (S2)
S3	91.67194444	29.81500000	3790	Sediment 3 (S3)
S4	91.20055556	29.65944444	3664	Sediment 4 (S4)
S5	91.14527778	29.64277778	3657	Sediment 5 (S5)
S6	91.07805556	29.64916667	3647	Sediment 6 (S6)
S7	90.99388889	29.58833333	3633	Sediment 7 (S7)
S8	90.93000000	29.44527778	3603	Sediment 8 (S8)
S9	90.85416667	29.36694444	3590	Sediment 9 (S9)
S10	90.77944444	29.35527778	3583	Sediment 10 (S10)

Table S2. The content of heavy metals in sediment from the Lhasa River ($mg \cdot kg^{-1}$). In this study, the sediment samples were freeze-dried before digestion. 0.1 g of dried sediment samples were put into tubes and added 4 mL HNO_3 and 2 mL HF, and then heated in a microwave at 150°C for 2h. After that, 30 mL of deionized water was added. The solution was filtered and stored at 4°C. The concentration of Cr, Co, Cu, Zn, Cd, Hg, Pb, and As in water, sediment, and plastic samples were analyzed with Inductively Coupled Plasma-Mass Spectrometry (ICP-MS). The detailed data was as follows:

	Cr	Co	Cu	Zn	Cd	Hg	Pb	As
**LSR1**	9.14	4.27	49.16	217.95	0.96	0.08	152.70	269.35
**LSR2**	31.81	5.64	21.33	350.75	0.70	0.03	171.10	798.50
**LSR3**	25.86	4.86	65.90	308.50	0.64	0.09	173.25	708.00
**LSR4**	22.42	4.24	107.10	360.25	3.37	0.05	63.35	732.50
**LSR5**	18.26	3.36	48.36	224.85	0.59	0.03	141.10	544.00
**LSR6**	14.03	3.07	33.75	229.45	0.40	0.02	106.40	554.00
**LSR7**	15.57	3.62	24.20	217.00	1.18	0.04	165.85	474.25
**LSR8**	14.73	2.92	25.07	242.70	0.87	0.02	135.90	573.00
**LSR9**	22.58	3.96	41.57	265.55	2.42	0.06	47.58	559.00
**LSR10**	15.23	3.32	43.63	215.40	0.50	0.03	101.90	524.50

Table S3. Sediment Physicochemical properties and nutrients of the Lhasa River.

	T	pH	Salinity (%)	Conductivity	TN (g/kg)	TC (g/kg)	TN/TC
S1	7.6	8.55	0.05	174	0.38	0.21	1.81
S2	13.1	8.58	0.06	161	0.74	0.18	4.02
S3	13.9	8.18	0.10	269	0.73	0.14	5.18
S4	15.9	8.43	0.03	110	0.33	0.12	2.68
S5	15.6	8.55	0.03	84	0.25	0.14	1.85
S6	16.4	8.77	0.04	130	0.28	0.18	1.54
S7	17.1	8.24	0.04	136	0.36	0.65	0.56
S8	15.3	8.35	0.05	141	0.17	0.08	2.27
S9	15.3	8.36	0.01	86	0.22	0.13	1.73
S10	16.5	8.26	0.03	104	0.32	0.24	1.33

Table S4. The primers sequence of ARGs and MGEs.

Target genes	Sequence	
	Primer F	Primer R
ARGs		
bla$_{TEM}$	5′- AGCATCTTACGGATGGCATGA -3′	5′- TCCTCCGATCGTTGTCAGAAGT-3′
bla$_{CMY}$	5′- CCGCGGCGAAATTAAGC -3′	5′- GCCACTGTTTGCCTGTCAGTT -3′
bla$_{CTX-M}$	5′- GGAGGCGTGACGGCTTTT -3′	5′- ACACCAGTGACAATATCACCG -3′
bla$_{NMD-1}$	5′- TTGGCCTTGCTGTCCTTG -3′	5′- ACACCAGTGACAATATCACCG -3′
blaCTX-M$_{-32}$	5′- CGTCACGCTGTTGTTAGGAA -3′	5′- CGCTCATCAGCACGATAAAG -3′
*aad*A	5′- GTTGTGCACGACGACATCATT -3′	5′- GGCTCGAAGATACCTGCAAGAA -3′
*str*B	5′- GCTCGGTCGTGAGAACAATCT -3′	5′- CAATTTCGGTCGCCTGGTAGT -3′
*sul*1	5′- CACCGGAAACATCGCTGCA -3′	5′- AAGTTCCGCCGCAAGGCT -3′
*sul*2	5′- CTCCGATGGAGGCCGGTAT -3′	5′- GGGAATGCCATCTGCCTTGA -3′
*sul*3	5′- TCCGTTCAGCGAATTGGTGCAG -3′	5′- TTCGTTCACGCCTTACACCAGC -3′
*tet*A	5′- GCTGTTTGTTCTGCCGGAAA -3′	5′- GGTTAAGTTCCTTGAACGCAAACT -3′
*tet*M	5′- CATCATAGACACGCCAGGACATAT -3′	5′- CGCCATCTTTTGCAGAAATCA -3′
*tet*X	5′- AAATTTGTTACCGACACGGAAGTT -3′	5′- CATAGCTGAAAAAATCCAGGACAGTT -3′
*ere*A	5′- CCTGTGGTACGGAGAATTCATGT -3′	5′- ACCGCATTCGCTTTGCTT -3′
*ere*B	5′- GCTTTATTTCAGGAGGCGGAAT -3′	5′- TTTTAAATGCCACAGCACAGAATC -3′
*qnr*A	5′- AGGATTTCTCACGCCAGGATT -3′	5′- CCGCTTTCAATGAAACTGCAA -3′
*qnr*B	5′- GGMATHGAAATTCGCCACTG -3′	5′- TTYGCBGYYCGCCAGTCG -3′
*qnr*S	5′- GTGAGTAATCGTATGTACTTTTGC -3′	5′- AAACACCTCGACTTAAGTCT -3′

*arm*A	5′- TGGGGGTCTTACTATTCTGCC -3′	5′- TGCGACTCTTTCATTCGTCG -3′
*mph*A	5′- CTGACGCGCTCCGTGTT -3′	5′- GGTGGTGCATGGCGATCT -3′
mcr-1	5′- CGGTCAGTCCGTTTGTTC -3′	5′- CTTGGTCGGTCTGTAGGG -3′
mcr-3	5′- AGAACGATGGAGGCTGCAAA -3′	5′- GCCAACCAGCTTATCCCCTT -3′
mcr-7	5′- CTCTCTGATCACGGCGAGTC -3′	5′- CTGCAGGCAGTTCAGATCCA -3′
MGEs		
*int*I1	5′- CGAACGAGTGGCGGAGGGTG -3′	5′- TACCCGAGAGCTTGGCACCCA -3′
*tnp*A	5′- AATTGATGCGGACGGCTTAA -3′	5′- TCACCAAACTGTTTATGGAGTCGTT -3′
Internal reference gene		
16S rRNA	5′- AGAGTTTGATCMTGGCTCAG -3′	5′- GWATTACCGCGGCKGCTG -3′

Table S5. Mantel tests of sediment physicochemical properties and nutrients factors against community similarity of abundant and rare bacteria.

	Community similarity		Phylogenetic similarity	
	Abundant	Rare	Abundant	Rare
Temperature	0.11	-0.079	-0.17	-0.284
pH	-0.025	0.137	0.119	0.083
Salinity	-0.347*	-0.039	-0.039	-0.129
Conductivity	-0.353*	-0.075	-0.092	-0.196
TN	-0.259	-0.145	-0.164	-0.282
TC	0.038	-0.095	-0.039	0.023
Cr	-0.036	-0.114	-0.082	-0.256
Co	-0.113	-0.077	-0.111	-0.213
Cu	-0.362*	-0.719**	-0.569**	-0.637**
Zn	-0.237	-0.429**	-0.356*	-0.478**
Cd	0.02	-0.467**	-0.467**	-0.405**
Hg	-0.189	0.037	-0.082	-0.125
Pb	0.09	-0.256	-0.216	-0.195
As	-0.114	-0.316*	-.310*	-0.493**

Table S6. Mantel tests of environmental variables and geospatial factors against βNTI of abundant and rare bacteria in the sediment of the Lhasa River.

	Abundant	Rare
Latitude	0.333*	0.334*
Longitude	0.131	0.115
Altitude	-0.126	-0.235
Temperature	-0.182	-0.246
pH	.453**	0.088
Salinity	0.281	0.192
Conductivity	.319*	0.186
TN	0.278	0.267
TN/TC ratio	.362*	0.216
TC	-0.058	0.111
Cr	0.018	-0.091
Co	0.102	0.103
Cu	-0.284	-0.047
Zn	-0.093	-0.112
Cd	-.466**	-0.114
Hg	0.049	-0.121
Pb	-0.224	0.159
As	-0.137	-0.241

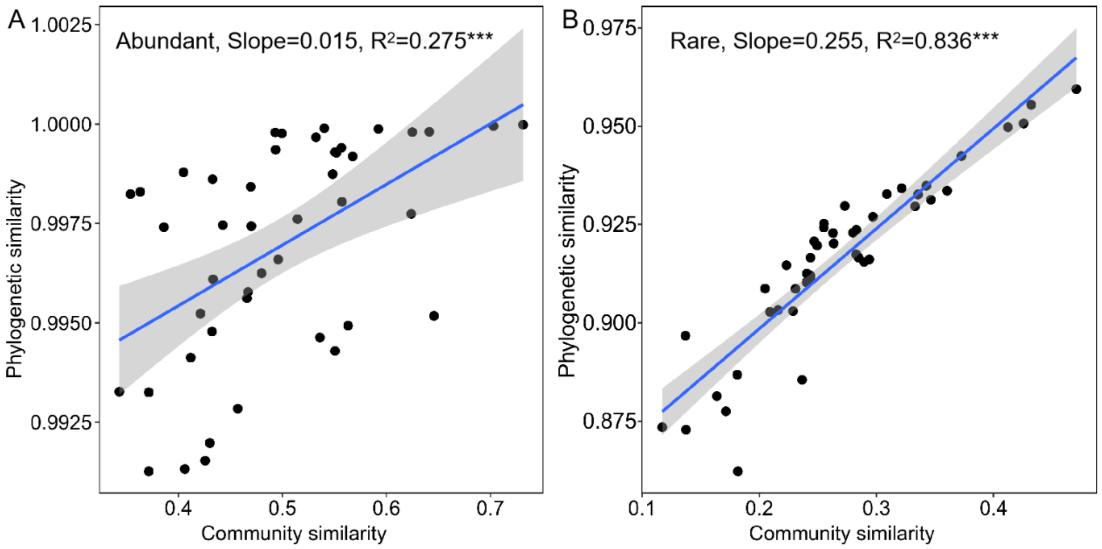

Fig. S1. Relationships between community similarity and phylogenetic similarity of abundant (A) and rare bacteria (B) in sediment from the Lhasa River. Asterisks represent the significance level (***, *P*-value < 0.001).

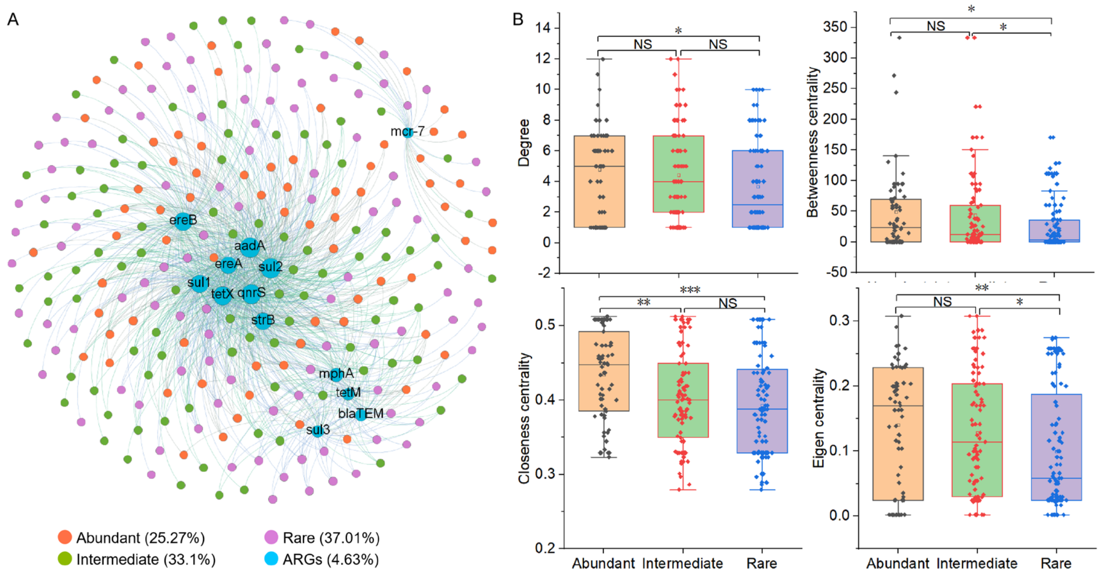

Fig. S2. Potential hosts of ARGs in the sediment of the Lhasa River. (A) Network analysis showed the potential hosts of ARGs, the percentages were these taxa OTUs or genes that accounted for total OTUs or genes in networks. A connection based on a strong (r > 0.8) and significant ($p < 0.01$) correlation via Spearman. (B) Showed main network topological properties of potential hosts of ARGs. Asterisks denote significance (NS, $p \geq 0.05$; *, $p < 0.05$; **, $p < 0.01$; ***, $p < 0.001$).

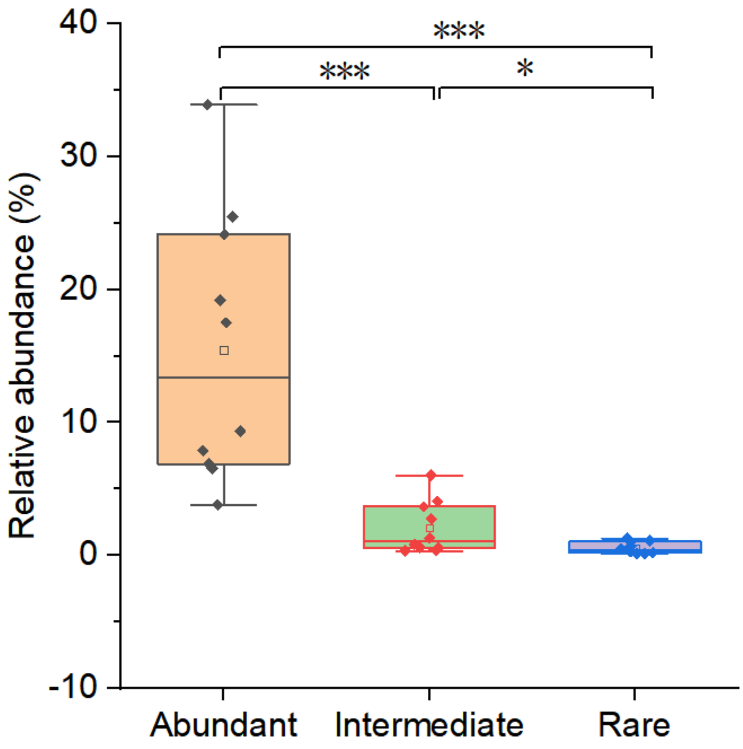

Fig. S3. Relative abundance of potential hosts of ARGs belonging to abundant and rare bacteria. Asterisks denote significance (*, $p < 0.05$; ***, $p < 0.001$).

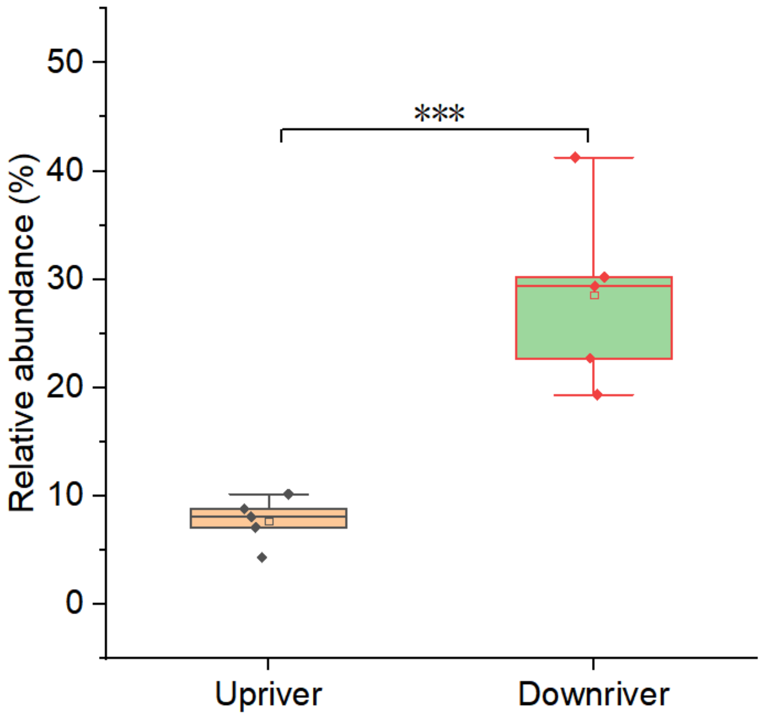

Fig. S4. Geospatial distribution of potential hosts of ARGs in the sediment of the Lhasa River. Upriver including the sediment samples from sampling site S1 to S5, and downriver including the sediment samples from sampling site S6 to S10. Asterisks denote significance (***, $p < 0.001$).

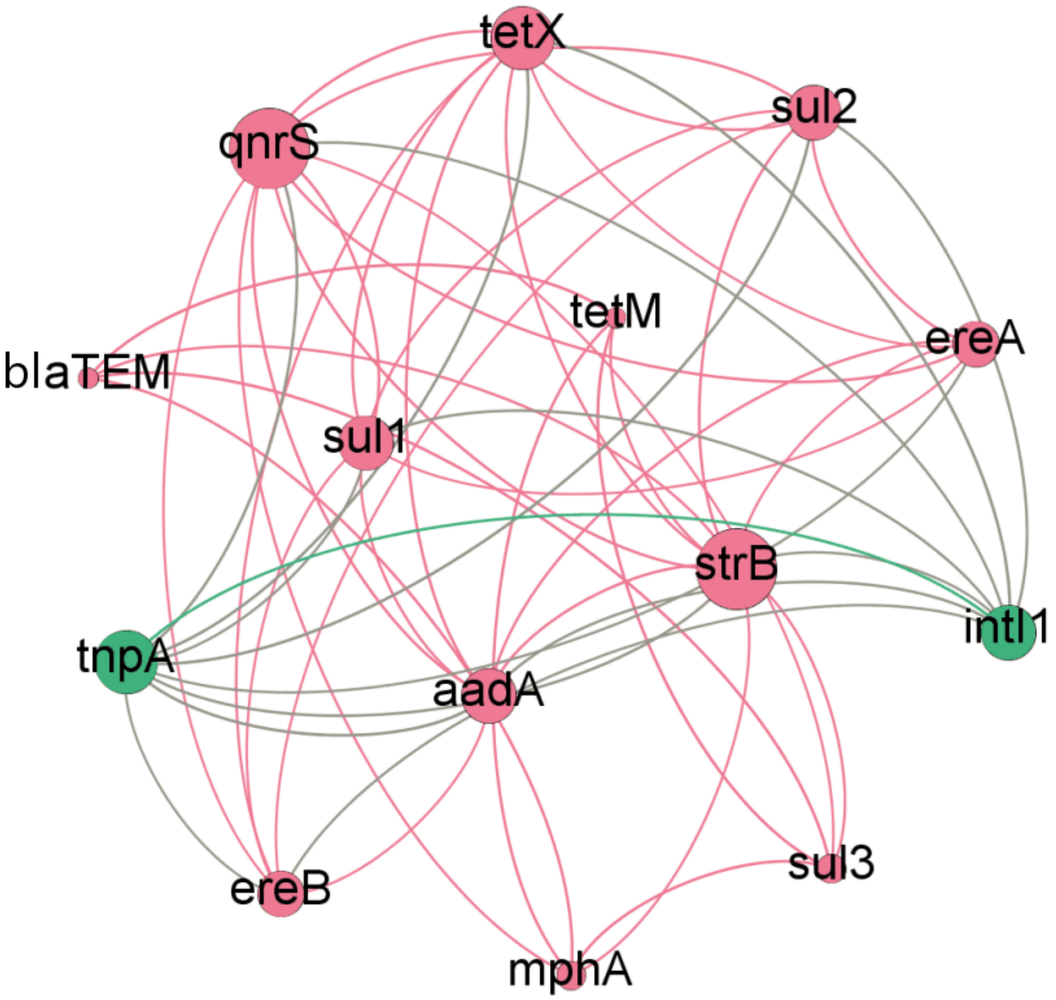

Fig. S5. Co-occurrence patterns of ARGs in the sediment of the Lhasa River. A connection based on a strong (r > 0.8) and significant ($p < 0.01$) correlation via Spearman.
